# Supplementary material for: Proadrenomedullin in the Management of COVID-19 Critically Ill Patients in Intensive Care Unit: A Systematic Review and Meta-Analysis of Evidence and Uncertainties in Existing Literature
Source: J Clin Med. 2022 Aug 4;11(15):4543. doi: 10.3390/jcm11154543 (PMC9369672; doi:10.3390/jcm11154543)
Supplement: Supplementary file 1 [file jcm-11-04543-s001.zip › jcm-1808239-supplementary.pdf]

Table S1.

Full name	Short name	Biochemical characteristics	Functional characteristics	Biomarker pitfall	Citations
precursor pro-hormone of ADM–mid regional pro-adrenomedullin	MR-proADM	longer half-life than ADM	prognostic value in disease	no known action by itself	Marino R. et al. 10.1186/cc13731.
bioactive adrenomedullin	bioADM	active, short-lived circulating peptide	associated with severe organ dysfunction and an elevated mortality risk	less used in the literature	Laterre PF et al. 10.1007/s00134-021-06537-5
plasma adrenomedullin	ADM	vasodilatory peptide	prognostic value in disease	short half-life	Marino R. et al. 10.1186/cc13731.

References

1. Marino R, Struck J, Maisel AS, Magrini L, Bergmann A, Di Somma S. Plasma adrenomedullin is associated with short-term mortality and vasopressor requirement in patients admitted with sepsis. *Crit Care*. 2014;18(1): R34. Published 2014 Feb 17. doi:10.1186/cc13731
2. Laterre PF, Pickkers P, Marx G, et al. Safety and tolerability of non-neutralizing adrenomedullin antibody adrecizumab (HAM8101) in septic shock patients: the AdrenOSS-2 phase 2a biomarker-guided trial. *Intensive Care Med*. 2021;47(11):1284-1294. doi:10.1007/s00134-021-06537-5
